# Informal Caregiving in Amyotrophic Lateral Sclerosis (ALS): A High Caregiver Burden and Drastic Consequences on Caregivers’ Lives

**DOI:** 10.3390/brainsci11060748

**Published:** 2021-06-04

**Authors:** Pavel Schischlevskij, Isabell Cordts, René Günther, Benjamin Stolte, Daniel Zeller, Carsten Schröter, Ute Weyen, Martin Regensburger, Joachim Wolf, Ilka Schneider, Andreas Hermann, Moritz Metelmann, Zacharias Kohl, Ralf A. Linker, Jan Christoph Koch, Claudia Stendel, Lars H. Müschen, Alma Osmanovic, Camilla Binz, Thomas Klopstock, Johannes Dorst, Albert C. Ludolph, Matthias Boentert, Tim Hagenacker, Marcus Deschauer, Paul Lingor, Susanne Petri, Olivia Schreiber-Katz

**Affiliations:** 1 Department of Neurology, Hannover Medical School, 30625 Hannover, Germany; Schischlevskij.Pavel@mh-hannover.de (P.S.); Mueschen.Lars@mh-hannover.de (L.H.M.); dr.almaosmanovic@gmail.com (A.O.); Binz.Camilla@mh-hannover.de (C.B.); Petri.Susanne@mh-hannover.de (S.P.); 2 Department of Neurology, Klinikum Rechts der Isar, Technical University of Munich, 81675 Munich, Germany; isabell.cordts@tum.de (I.C.); marcus.deschauer@mri.tum.de (M.D.); paul.lingor@tum.de (P.L.); 3 Department of Neurology, University Hospital Carl Gustav Carus, Technische Universität Dresden, 01307 Dresden, Germany; Rene.Guenther@uniklinikum-dresden.de; 4 German Center for Neurodegenerative Diseases (DZNE), 01307 Dresden, Germany; 5 Department of Neurology, University Medicine Essen, 45147 Essen, Germany; benjamin.stolte@uk-essen.de (B.S.); tim.hagenacker@uk-essen.de (T.H.); 6 Department of Neurology, University of Würzburg, 97080 Würzburg, Germany; Zeller_D@ukw.de; 7 Hoher Meißner Clinic, Neurology, 37242 Bad Sooden-Allendorf, Germany; Schroeter@reha-klinik.de; 8 Department of Neurology, Ruhr-University Bochum, BG-Kliniken Bergmannsheil, 44789 Bochum, Germany; ute.weyen@bergmannsheil.de; 9 Department of Molecular Neurology, Friedrich-Alexander-University Erlangen-Nürnberg, 91054 Erlangen, Germany; Martin.Regensburger@uk-erlangen.de; 10 Department of Neurology, Diakonissen Hospital Mannheim, 68163 Mannheim, Germany; j.wolf@diako-mannheim.de; 11 Department of Neurology, Martin-Luther University Halle/Saale, 06120 Halle, Germany; Ilka.Schneider@sanktgeorg.de; 12 Department of Neurology, Klinikum Sankt Georg, 04129 Leipzig, Germany; 13 Translational Neurodegeneration Section “Albrecht-Kossel”, Department of Neurology, University Medical Center Rostock, University of Rostock, 18147 Rostock, Germany; Andreas.Hermann@med.uni-rostock.de; 14 German Center for Neurodegenerative Diseases (DZNE), Rostock/Greifswald, 18147 Rostock, Germany; 15 Department of Neurology, University Hospital Leipzig, 04103 Leipzig, Germany; Moritz.Metelmann@medizin.uni-leipzig.de; 16 Department of Neurology, University of Regensburg, 93053 Regensburg, Germany; zacharias.kohl@klinik.uni-regensburg.de (Z.K.); Ralf.Linker@klinik.uni-regensburg.de (R.A.L.); 17 Department of Neurology, University Medicine Göttingen, 37075 Göttingen, Germany; jkoch@med.uni-goettingen.de; 18 Department of Neurology, Friedrich-Baur Institute, University Hospital, Ludwig Maximilian University of Munich, 80336 Munich, Germany; Claudia.Stendel@med.uni-muenchen.de (C.S.); Thomas.Klopstock@med.uni-muenchen.de (T.K.); 19 German Center for Neurodegenerative Diseases (DZNE), 80336 Munich, Germany; 20 Munich Cluster for Systems Neurology (SyNergy), 80336 Munich, Germany; 21 Department of Neurology, University of Ulm, 89081 Ulm, Germany; johannes.dorst@rku.de (J.D.); albert.ludolph@rku.de (A.C.L.); 22 German Center for Neurodegenerative Diseases (DZNE), 89081 Ulm, Germany; 23 Department of Neurology, Institute of Translational Neurology, University Hospital Münster, 48149 Münster, Germany; Matthias.Boentert@ukmuenster.de; 24 Department of Medicine, UKM Marienhospital, 48565 Steinfurt, Germany

**Keywords:** amyotrophic lateral sclerosis (ALS), informal caregiving, caregiver burden, functional status, decreasing autonomy, depression, anxiety, health-related quality of life, socioeconomic status, psychological support

## Abstract

Amyotrophic lateral sclerosis (ALS) is a fatal neurodegenerative disease that causes progressive autonomy loss and need for care. This does not only affect patients themselves, but also the patients’ informal caregivers (CGs) in their health, personal and professional lives. The big efforts of this multi-center study were not only to evaluate the caregivers’ burden and to identify its predictors, but it also should provide a specific understanding of the needs of ALS patients’ CGs and fill the gap of knowledge on their personal and work lives. Using standardized questionnaires, primary data from patients and their main informal CGs (*n* = 249) were collected. Patients’ functional status and disease severity were evaluated using the Barthel Index, the revised Amyotrophic Lateral Sclerosis Functional Rating Scale (ALSFRS-R) and the King’s Stages for ALS. The caregivers’ burden was recorded by the Zarit Burden Interview (ZBI). Comorbid anxiety and depression of caregivers were assessed by the Hospital Anxiety and Depression Scale. Additionally, the EuroQol Five Dimension Five Level Scale evaluated their health-related quality of life. The caregivers’ burden was high (mean ZBI = 26/88, 0 = no burden, ≥24 = highly burdened) and correlated with patients’ functional status (r_p_ = −0.555, *p* < 0.001, *n =* 242). It was influenced by the CGs’ own mental health issues due to caregiving (+11.36, 95% CI [6.84; 15.87], *p* < 0.001), patients’ wheelchair dependency (+9.30, 95% CI [5.94; 12.66], *p* < 0.001) and was interrelated with the CGs’ depression (r_p_ = 0.627, *p* < 0.001, *n =* 234), anxiety (r_p_ = 0.550, *p* < 0.001, *n =* 234), and poorer physical condition (r_p_ = −0.362, *p* < 0.001, *n =* 237). Moreover, female CGs showed symptoms of anxiety more often, which also correlated with the patients’ impairment in daily routine (r_s_ = −0.280, *p* < 0.001, *n =* 169). As increasing disease severity, along with decreasing autonomy, was the main predictor of caregiver burden and showed to create relevant (negative) implications on CGs’ lives, patient care and supportive therapies should address this issue. Moreover, in order to preserve the mental and physical health of the CGs, new concepts of care have to focus on both, on not only patients but also their CGs and gender-associated specific issues. As caregiving in ALS also significantly influences the socioeconomic status by restrictions in CGs’ work lives and income, and the main reported needs being lack of psychological support and a high bureaucracy, the situation of CGs needs more attention. Apart from their own multi-disciplinary medical and psychological care, more support in care and patient management issues is required.

## 1. Introduction

Amyotrophic lateral sclerosis (ALS) is a devastating neurodegenerative condition characterized by loss of upper and lower motor neurons that results in atrophy and weakness of bulbar, limb and trunk muscles, and frequently culminates in severe respiratory failure leading to death within two to five years after symptom onset [1,2]. Approved disease-modifying therapies only marginally delay symptom progression [3,4]. This fatal condition puts a significant burden not only on the affected patient but also on her/his family and caregiver (CG) [5]. Patients’ dependence and the need for care inevitably increase with symptom progression. A considerable proportion of this care is provided by informal CGs who are mostly partners or close relatives [6,7]. 

Meanwhile, caregiving in ALS is of high interest and several studies addressed different aspects of this topic. Over the course of the last two decades of the last century, the role of family caregivers and the integration of care for patients and their families were investigated [8,9], especially in the context of home care and ventilation [10,11,12]. In the 2000s, more frequent longitudinal analyses of the ALS-associated caregiver burden advanced [13,14,15]. Caregiving in ALS turned into an international topic of interest, which cumulated in numerous literature reviews [16,17,18]. During the last years, new information technology, even machine learning approaches, were used to predict caregiver burden [19]. Although, there is a lot of knowledge on caregiving in ALS and the needs of CGs [20], established institutional structures to support informal caregivers and the families of the affected patients are still missing, as far as we know, in Germany and other countries. There are investigations of different strategies to relieve the families’ caregiver burden in neurological disorders, like paid CGs instead of informal CGs and mobile gerontopsychiatric counseling services in dementia [21,22], psychological support for partners of patients with ALS [23], psychoeducational intervention in people with Parkinson’s disease and their informal caregivers [24], or even new technologies like the use of a brain-computer interfaces-based home care system [25]. Nevertheless, none of these showed a widespread success, so as not to be implemented into standard care yet; therefore, unmet needs commonly remain unchanged.

A high caregiver burden has previously been demonstrated [26,27] and different patient-related predictors such as assisted ventilation [6,28,29], or CG-related factors like anxiety and depression [13,15,27] have been identified. In contrast, factors such as mindfulness were shown to reduce caregiver burden [30]. While there is no unanimous consensus in the correlation of caregiver burden and the patient’s functional status [14,27,31,32], it has clearly been demonstrated that nursing worsens CGs’ quality of life [14,15,16,33,34], and is associated with anxiety and depression [15,34,35]. The interrelation between these parameters has so far only been touched superficially [16] and is mainly considered to be a chain of effects. Moreover, current German data in a greater patient cohort are still missing, and most studies did not specifically investigate the effects of caregiving on the CGs’ working lives and socioeconomic status. In addition, there are some unresolved questions regarding gender influence on caregiver burden. Women, who are most likely the primary CGs in ALS, were reported to show a higher burden, especially in psychosocial dimensions [36] and to be at higher risk for depressive symptoms [37]. In contrast, some other studies neither observed any effect of CGs’ gender on burden scores [14,33] nor on quality of life or depression according to the patients’ or CGs’ gender [27,35,38]. With these questions still unanswered, the effort of this Germany-wide multi-center study was to provide evidence for understanding the needs of CGs in ALS and shed more light on this complex and important topic. We used different surveys not only to measure burden (Zarit Burden Interview, ZBI) and duration of caregiving (DOC, in hours per day, h/day) as dependent variables but also examined its predictors from both patients’ (e.g., (exempli gratia), age, gender, functional status and disease severity) and CGs’ characteristics (e.g., age, gender, own health status). Moreover, we aimed to evaluate the impact of nursing on the main CGs’ individual lives from the various perspectives: quality of life (QoL), depression and anxiety, physical and mental health, daily routine, working situation, and wishes and needs. 

## 2. Materials and Methods

### 2.1. Patient Recruitment

The data for this prospective, cross-sectional, multi-center study were collected between August 2018 and March 2020 within a Germany-wide assessment of disease-related costs, quality of life and caregiver burden [39]. The El Escorial diagnostic criteria for ALS [40] were used to enroll adult (≥18 years old) patients with clinically possible, probable (incl. laboratory supported) and definite ALS with their main CGs. Patient recruitment took place at 17 specialized motor neuron disease (MND) clinics, which are members of the MND Network (German Network for Motor Neuron Diseases) [41,42]: Hannover (principal investigator); Munich (Klinikum rechts der Isar, Technical University of Munich, and Friedrich-Baur Institute, Ludwig Maximilian University of Munich); Dresden; Würzburg; Bad Sooden-Allendorf; Bochum; Erlangen; Ulm; Mannheim; Halle/Saale; Rostock; Leipzig; Regensburg; Göttingen; Münster; and Essen. Patients with a permanent residence outside of Germany and patients who could not adequately respond to the questionnaire (e.g., due to severe cognitive impairment or language problems) were excluded. However, in severely affected ALS patients, help by a proxy to answer the questionnaire was allowed. In order to reduce selection bias, all patients who visited the above-named clinics during the recruitment period that met the inclusion criteria were identified as potentially eligible (*n* = 1147). The total response rate was 35.2% (404/1147). Self-reported reasons to refuse participation in this study were the expenditure of time or participation in another study. Patients and CGs either answered the paper-based study questionnaires anonymously or gave their written informed consent to contribute data in a pseudonymous way. 

This study report was structured following the reporting guidelines of Strengthening the Reporting of Observational Studies in Epidemiology [43].

### 2.2. Data Collection and Assessment Instruments

Pairs of patients and their main informal CGs answered standardized self-designed questionnaires by hand, similar to those previously described in disease burden studies by the authors [7,39,44,45]. In addition to baseline demographics and disease history, patients and CGs reported on the patients’ functional status. Furthermore, CGs’ employment data, caregiving, health impairment and other nursing repercussions were evaluated. The focus of this study was the person, whom the patient identified as her/his main informal CG. This did not exclude that more than one person, if necessary, may have cared for the patient (e.g., in case of permanent supervision). However, the study’s aim was the investigation of the main informal CG’s individual life. Professional CGs were not taken into account.

#### 2.2.1. Patients’ Functional Status

The Barthel Index (BI) [46] is a valid instrument to measure physical disability and is frequently used in the context of rehabilitation [47]. It has also previously been used in ALS [48,49]. In this study, the functional status of the patient’s during daily life was proxy-reported by the CG by using this measure. The BI contains ten questions, which describe the approximated dependence of a person in different activities of daily living (feeding, bathing, grooming, dressing, bowel and bladder care, toileting, bed and chair transfer, general mobility, and climbing stairs). Each performance is rated with 0/5/10 and some with up to 15 points (bed and chair transfer/general mobility). The sum of the ten items produces a score from 0–100 points. Patients can be categorized into four groups: a total BI score of 0–20 implies full dependence (group 1); 21–60 moderate dependence (group 2); 61–99 slight dependence (group 3); and 100 points (group 4) are associated with full independence from others [46,50]. In case of missing data from one question, the individual BI score’s arithmetic mean was applied.

The second measurement to record the patient’s self-reported functional status was the revised Amyotrophic Lateral Sclerosis Functional Rating Scale (ALSFRS-R), an approved clinical measure of functional impairment and symptoms in ALS [51,52]. The scale comprises four domains that enclose gross motor function, fine motor function, bulbar function, and respiratory function within twelve different items rated with 0–4 points. By adding these up, the minimum of 0 points implies full dependence of a person and the maximum of 48 points equals no impairment [53]. In case of missing data from one question in one distinct domain, the arithmetic mean points of that patient in that domain were applied.

In our study, the BI and ALSFRS-R determined the patient’s functional status from a proxy- and a self-reported perspective and respectively her/his dependence on the CG. The surveys were used as independent variables in several analyses (e.g., caregiver burden, general, health and working life impairment). Although both instruments record a patient’s functional status, the BI focuses on nursing-related questions. For this reason, it was assessed by the CG in contrast to the ALSFRS-R, which was evaluated by the patients themselves in order to compare the reports’ differences.

#### 2.2.2. Disease Severity

To provide a supplementary analysis, the King’s Clinical Staging System for ALS [54] was used to subdivide the patients into five groups. To determine the King’s Stages, a derivation from the ALSFRS-R [52] was applied. Continuous clinical involvement of central nervous system regions throughout the course of the disease is categorized. Each stage correlates with the number of anatomical regions affected, while stage 1 implies one affected region; stage 2 two regions; stage 3 three regions; stage 4A coincides with the need for a gastrostomy due to nutritional failure; stage 4B implies the need for assisted ventilation due to respiratory failure; and stage 5 being fatal. In this study, the King’s Stages determined the patient’s disease severity and were used as an independent variable in several analyses (e.g., caregiver burden, general, health and working life impairment) in order to verify its role as a predictor of caregivers’ burden. 

#### 2.2.3. Caregivers’ Burden and Duration of Care

The Zarit Burden Interview (ZBI) [55] quantified the caregivers’ burden. The ZBI is a commonly used method to measure caregivers’ burden in ALS and other neurological diseases [56] and was validated in its German version, e.g., for dementia [57]. The questionnaire consists of 22 items, which evaluate the effect of caregiving on the personal and social lives of CGs, their psychological burden and feelings of guilt. Each question is scored from 0–4 points on a response scale, resulting in a range from 0 points (no burden) to 88 points (highest burden) [58]. As previously suggested, values ≥ 24 are considered to reflect a high burden [59], so that we defined a “low burden group” (ZBI < 24) and a “high burden group” (ZBI ≥ 24) for further analysis. In our study, the ZBI determined the caregiver burden and was used as a dependent variable in several correlation analyses and as an independent variable in the low vs. high burden group comparison. In case of missing data from one question, the CG’s individual arithmetic mean of the ZBI score was applied.

The duration of care (DOC) was investigated as another dependent variable, on which the influence of other (patient- and CG-associated) factors was analyzed. In our self-designed question, the interviewed CGs stated their hours of caregiving and support of the patients per day. Nonetheless, the patient might have demanded more care (e.g., permanent supervision) which was provided by other (secondary) CGs. We did not assess this additional time, since the study focused on the main CG’s individual life.

#### 2.2.4. Caregivers’ Anxiety and Depression

The Hospital Anxiety and Depression Scale (HADS) determines symptoms of anxiety and depression. The HADS is a well performing and valid instrument to measure anxiety disorder and depression in general populations [60] and was previously used in caregiver burden analyses in ALS [35]. Its advantage over other instruments is the ability to measure both anxiety and depression. The questionnaire consists of 14 items, which are scored with 0–3 points. Seven items each correspond to the anxiety subscale (HADS-A) and the depression subscale (HADS-D) with a range from 0–21 points respectively [61]. Values that equal or exceed 8 points ensure an optimal balance between specificity and sensitivity in the implication of the presence of anxiety or depression. In our study, the HADS was used to describe the influence of caregiving on the CGs’ lives and to quantify the occurrence of anxiety and depression under CGs of ALS-affected patients. In case of missing data from one question, the participant was excluded from any analyses related to this score.

#### 2.2.5. Caregivers’ Health-Related Quality of Life

To evaluate the health-related quality of life (HRQoL), which may also be deteriorated through caregiving, the German version of the EuroQol Five Dimension Five Level Scale (EQ-5D-5L) was utilized [62]. This instrument is commonly used in the general population and CG analyses in ALS [34,63,64]. The EQ-5D-5L consists of five questions in five different domains: mobility, self-care, usual activities, pain/discomfort, and anxiety/depression. Every domain is rated in five stages from 1 (no problems) to 5 (severe problems) forming distinct health states (from 11,111 to 55,555). Following the provider’s instruction, the resulting health state can be converted into an index value (EQ-5D-5L index value, from <0 (worse than dead) to 1.0 (full health)), using a German value set [65]. Furthermore, a visual analog scale (EQ-VAS; from 0 (worst) to 100 (best imaginable health today)) was used to record the self-rated current health. Since one of its domains deals with anxiety/depression, there is an overlap with the HADS. However, the EQ-5D-5L does not differentiate between anxiety and depression, because it uses an “or” in its’ question. Therefore, with the EQ-5D-5L it remains unknown if the CG suffers from depression or anxiety or both. For this reason, the HADS was additionally used to evaluate depression and anxiety in our analysis. In the case of missing data in one domain, the participant was excluded from any analyses related to HRQoL.

#### 2.2.6. Caregivers’ General Impairment in Daily Routine

In our self-developed question, the CGs were able to indicate their perceived general impairment in everyday life on a four-point Likert scale from “not impaired at all” to “extremely impaired”. The domains considered were: time; physical; mental and social limitations; as well as cuts in general flexibility and mobility. The ratio of answers given on the Likert scale were used as a dependent variable in analyses according to the BI groups and CGs’ gender as independent variables in order to identify the influence of these independent variables on CGs’ general impairment that was associated to caregiving.

#### 2.2.7. Caregivers’ Work and Health Impairment, and Their Wishes and Needs

Moreover, to evaluate CGs’ health and working life impairment due to caregiving was an additional objective of this study. These domains were part of the standardized self-designed questionnaire that we used. More precisely, the individual health impairment due to caregiving was evaluated with questions such as “Do you suffer from physical and/or mental health problems due to the caregiving?”, which could be answered with the dichotomous answer “yes” or “no”. In the following question, physical symptoms like “back pain” or “knee pain” were stated by checking a list of preselected answers—the same was done for mental symptoms. 

Working life impairment due to caregiving was evaluated with different questions, e.g., “Did you have to change your workplace due to caregiving?” or “Did you have to change your working hours per week due to caregiving?”, which again were answered with a “yes” or “no”. The proportions of the answers were used as dependent variables in combination with the BI groups and CGs’ gender as independent variables. 

Further, the CGs were able to express their wishes and needs in a free text. All answers were categorized into superordinate categories. Answers such as “I had trouble to get an electric wheelchair for my husband because our health insurance wanted different specific documents” were categorized as “bureaucracy of health insurances”, and “We often do not have enough money since I stopped working” was categorized under “wish for financial support”. The number of answers in each category was counted to rank the categories depending on their importance to numerous CGs.

Finally, the CGs had the chance to rate their satisfaction with their own care in one question. The answers were 1 (very satisfied) to 4 (not satisfied at all) on a Likert scale. This question was investigated to roughly quantify their treatment satisfaction.

### 2.3. Statistical Analysis

The main data management and all analyses were performed at Hannover Medical School. Statistical analysis was performed using IBM^®^ Statistical Software Package of Social Science (SPSS^®^, IBM, Armonk, NY, USA) version 26. Descriptive statistics were calculated and depicted as percentage (%), mean and standard deviation (SD) or median and interquartile range (IQR). Normal Gaussian data distribution was tested using the Shapiro–Wilk and Kolmogorov–Smirnov test. The Mann–Whitney test was used to compare arithmetic mean differences between two groups (ZBI low vs. (versus) highly burdened, gender analysis, abnormally distributed data). ANOVA in combination with Bonferroni post-hoc analysis helped to compare arithmetic mean differences between more than two subgroups. As the total data did not appear to be normally distributed, an additional non-parametric test, the Kruskal–Wallis test (KWT) served as sensitivity analysis and revealed comparable results to the ANOVA. To determine the correlation between two metrically scaled variables, e.g., BI vs. ZBI, the Pearson correlation coefficient (r_p_) was used. The Spearman rank correlation coefficient (r_s_) determined a possible correlation between one ordinally and one metrically scaled variable, e.g., King’s Stages vs. HADS-D. To examine the correlation between ordinal and dichotomous variables, e.g., BI groups vs. drop in salary (yes/no), the χ^2^ (chi-square) test with Cramér’s V (φ_c_) were applied. Possible influencing factors on the caregivers’ burden were analyzed in a simple linear regression analysis. Hereby, the ZBI score and the DOC were defined as dependent variables and patient-related parameters (gender, age, disease onset, use of wheelchair, permanent attendance of a helper, use of a percutaneous endoscopic gastrostomy, use of ventilation support, employment) as well as caregiver-related parameters (gender, age, physical health impairment due to caregiving, mental health impairment due to caregiving, employment) were analyzed as possible influencing independent variables. If a variable turned out to be statistically significant (*p* ≤ 0.05), it was added into the multiple linear regression analysis (again, with the ZBI and the DOC as dependent variables). Significance levels were set at *p* ≤ 0.05 (two-tailed) for all analyses. In case of missing data from one of the measurements, cases were excluded only for this analysis, which resulted in different participant numbers within the depicted results. All statistical results should not be regarded as confirmatory, but rather as hypothesis generating. Due to the exploratory character of the study, we did not adjust for multiple testing. To control for center-bias, the patients’ functional status (BI, ALSFRS-R) and the caregiver burden (ZBI) outcomes were compared throughout the centers using ANOVA. No significant differences between the centers were observed. In addition, we gathered a representative Germany-wide ALS cohort regarding age, gender and the distribution of disease severity (see results) as well as a roughly matched regional distribution. 

## 3. Results

### 3.1. Study Population

From all over Germany, out of 404 returned questionnaires, 325 patients with ALS, corresponding to the inclusion criteria, were identified. Additionally, patients without a filled caregiver questionnaire were excluded from further analyses, resulting in 249 full datasets that were analyzed, including questionnaires completely answered by ALS patients and their main CGs. Data from both the patient’s and CG’s perspectives were evaluated and presented in this study.

Related to the estimated prevalence of ALS [66] in Germany, about 6600 patients may have lived with this diagnosis in 2019 [67]. Overall, we managed to include nearly 4% of them and their CGs for this study. The distribution of our patients roughly matched the population distribution by state in Germany, although patients from Lower Saxony were overrepresented (the study’s principal investigator was located at Hannover), and some states were underrepresented (Appendix A Table A1).

#### 3.1.1. Patients’ Characterization

Of the patients in this study, 64.1% were male, which matches previous findings that ALS is more prevalent in men [68]. The median age was 65 with a range from 27 to 88 years old. In general, the distribution of gender and age in our cohort appeared to be representative according to previous literature [69,70]. Most patients (89.3%) were married or in a partnership, and lived together with their partner (91.0%). About half of the patients (48.2%) used a wheelchair, 21.3% used home ventilator support, 15.3% were nourished using a percutaneous endoscopic gastrostomy (PEG), and 41.6% of patients required permanent (24 h) supervision. The median BI score was 60/100 and the median ALSFRS-R score was 32.5/48. No significant differences between men and women were observed, besides that female patients in our cohort showed a stronger impaired functional status (mean BI = 50.97/100, SD = 31.26, *n =* 88/mean ALSFRS-R = 29.61/48, SD = 9.33, *n =* 87) than men (mean BI = 62.58/100, SD = 30.79, *n =* 157/mean ALSFRS-R = 32.38/48, SD = 9.50, *n =* 157; BI, U = 5415.00; Z = −2.81; *p* = 0.005; *n =* 245/ALSFRS-R, U = 5593.00; Z = −2.34; *p* = 0.019; *n =* 244). The BI groups and the King’s Stages were well distributed, as the cohort included patients in all disease severity stages (Table 1).

#### 3.1.2. Caregivers’ Characterization

In the study, 69.9% of the CGs were female. The median age was 61 with a range from 18 to 88 years old. In our cohort, female CGs were younger than male CGs (U = 4693.00; Z = −3.46; *p* = 0.001; *n =* 248). Out of four possible patterns, in 61.2% the patient was male and the CG was female, in 26.9% the patient was female and the CG was male, in 9.0% the patient and the CG were female, and in 2.9% of the cases, the patient, as well as the CG were male. Most CGs (83.1%) were the patients’ spouses with whom they lived together, which reflects previous studies [6,27]. All but 0.8% (friends, *n =* 2) of the CGs were direct family members. While 8.0% had to stop working due to their partner’s condition and 47.8% were either retired, unemployed or a homemaker, 44.2% of the CGs were still employed and thus worthy of consideration for the work life impairment analysis. The median duration of caregiving (DOC) was three hours per day (as stated by the main CG; additional hours by more than one CG per patient are possible). 42.6% of the CGs reported an individual health deterioration due to caregiving. With a median ZBI score of 26/88, the cohort was highly burdened [59]. The EQ-VAS median with 75/100 was lower than the calculated EQ-5D-5L index value median with 0.909/1. The median HADS-D and HADS-A scores were 8/21 and 9/21, respectively, which indicated the presence of depression and anxiety. Besides higher anxiety in female CGs (see Section 3.6), no mentionable differences between men and women were observed (Table 2).

### 3.2. Caregivers’ General Burden in Relation to Patients’ Functional Status and Disease Severity

Since the ALSFRS-R score was self-reported by the patients and the BI a proxy-reported score by the CGs, both scores and their derivations (BI groups and King’s Stages) were used for data analysis in order to improve comparability.

**Figure 1:**
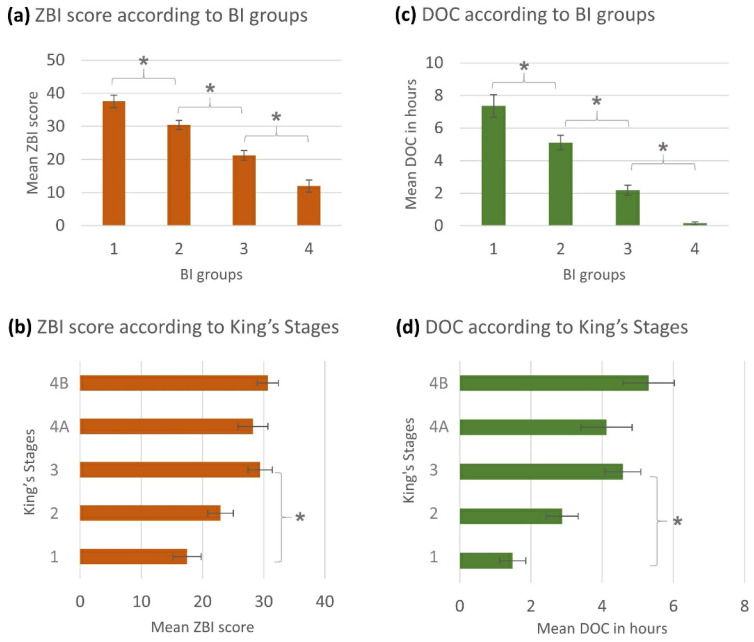
Evaluation of caregivers‘ general burden: Mean scores are presented with standard errors (error bars). Statistically significant findings: (**a**) ZBI-BI groups (ANOVA: *p* < 0.001; KWT: *p* < 0.001; r_s_ = −0.542, *p* < 0.001, *n =* 242), ZBI-BI (r_p_ = −0.555, *p* < 0.001, *n =* 242); (**b**) ZBI-King’s Stages (ANOVA: *p* < 0.001; KWT: *p* < 0.001; r_s_ = 0.300, *p* < 0.001, *n =* 237), ZBI-ALSFRS-R (r_p_ = −0.460, *p* < 0.001, *n =* 237); (**c**) DOC-BI groups (*p* < 0.001; KWT: *p* < 0.001; r_s_ = −0.636, *p* < 0.001, *n =* 249), DOC-BI (r_p_ = −0.617, *p* < 0.001, *n =* 249); (**d**) DOC-King’s Stages (*p* < 0.001; KWT: *p* < 0.001; r_s_ = 0.294, *p* < 0.001, *n =* 244) DOC-ALSFRS-R (r_p_ = −0.452, *p* < 0.001, *n =* 244). * = post-hoc: *p* ≤ 0.05 between subgroups. Abbreviations: ZBI = Zarit Burden Interview; BI = Barthel Index; DOC = duration of caregiving, ALSFRS-R = Revised Amyotrophic Lateral Sclerosis Functional Rating Scale.

The ANOVA showed statistically significant differences in the arithmetic means of the ZBI scores between the BI groups (Figure 1a) and the King’s Stages (Figure 1b). This means that an increase in patient’s functional impairment led to a higher caregiver burden. This result was supported by a positive correlation between the ZBI score and the King’s Stages (r_s_ = 0.300, *p* < 0.001, *n =* 237) and a negative correlation between the ZBI score and the ALSFRS-R (r_p_ = −0.460, *p* < 0.001, *n =* 237). Additionally, there was a strong negative correlation between the ZBI score and the BI score (r_p_ = −0.555, *p* < 0.001, *n =* 242).

The patient’s functional status did not only have an impact on the CG’s burden but also, as expected, on the DOC. We observed similar statistically significant differences in the arithmetic means of the DOC between the BI groups (Figure 1c) and the King’s Stages (Figure 1d). Thus, the higher the patients’ functional impairment was, the higher was the DOC. This finding was additionally supported by strong correlations between the DOC and the BI score (r_p_ = −0.617, *p* < 0.001, *n =* 249) as well as between the DOC and the King’s Stages (r_s_ = 0.294, *p* < 0.001, *n =* 244) and the ALSFRS-R (r_p_ = −0.452, *p* < 0.001, *n =* 244). Moreover, the analysis of the differently reported BI and ALSFRS-R scores revealed a comparable result of increasing ZBI and DOC in relation to the progressive patient’s autonomy loss, whereby the proxy-reported BI showed a stronger correlation to the dependent variables. 

### 3.3. Influencing Factors on Caregiver Burden

As already shown above, the patient’s functional status had a clear influence on her/his caregiver’s burden. To provide a deeper analysis of further influencing factors on caregiver burden (ZBI score), multiple regression analysis was used (Table 3). The patient’s wheelchair dependency increased the ZBI score by 9.30 points. Additionally, a rise of 5.01 points was observed, if the patient needed permanent supervision. The main influence on the ZBI appeared to be the CG’s mental health impairment due to caregiving with an increase of 11.36 points. However, physical health impairment had no statistically significant impact on the ZBI score. Furthermore, patients’ age seemed to slightly lower the burden by −0.24 points for each increasing year. Neither the patient’s nor the CG’s gender had statistically significant impacts on the ZBI in the multivariate regression analysis.

Further, we analyzed the influencing factors on the DOC. Again, the patient’s functional status had a deep impact (see above/Section 3.2). Moreover, it was observed that the need for permanent supervision increased the DOC by 1.86 h/day (Table 4). Additionally, a rise of 1.32 h/day was observed, if the patient needed ventilation support. The main influencing factor on the DOC appeared to be the wheelchair dependency of the patient with an increase of 2.29 h/day. Furthermore, the DOC rose by 1.62 h/day if the CG was suffering from physical health impairment due to caregiving. However, the CG’s mental health status had no statistically significant impact on the DOC.

Again, neither the patient’s nor the CG’s gender had statistically significant impacts on the DOC in the multivariate regression analysis.

### 3.4. Impact of Caregiving on the Caregivers

**Figure 2:**
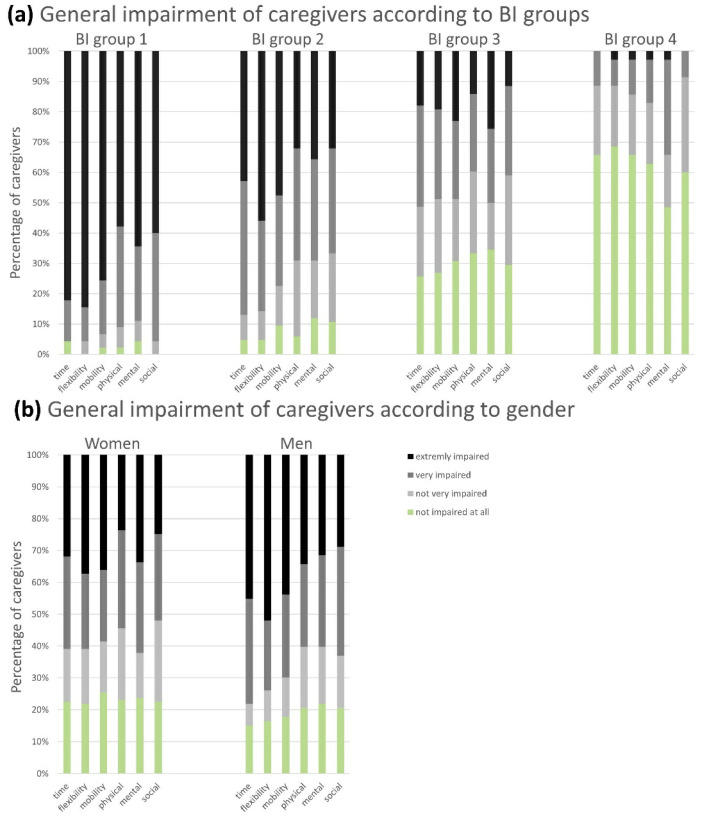
General impairment of caregivers according to BI groups (**a**) and gender (**b**): The caregivers were able to indicate their perceived general impairment in different dimensions of their everyday life on a four-point Likert scale from “not impaired at all” to “extremely impaired”. Six adjacent columns are assigned to one distinct BI group (**a**) or gender (**b**). One column corresponds to one domain. The extent of the colored sections represents the percentage of answers provided by the CGs on the abovementioned scale. The proportion of “very/extremely” impaired CGs increased with the loss of autonomy of the patient (BI group 4 (= BI score 100 points = full independence) to BI group 1 (= BI score 0–20 points = full dependence)), and the number of “not very/not at all” restricted CGs correspondingly decreased (**a**), while no significant differences were observed according to the CGs’ gender (**b**). Abbreviations: BI = Barthel Index; CGs = caregivers.

CGs stated their general impairment in daily routine on a Likert scale in six different domains: time, physical, mental, and social limitations, as well as cuts in general mobility and flexibility. The impairment decreased alongside the BI group increase (which meant a higher patient independency) in all domains (Figure 2a). That indicated that the perceived everyday impairment of the CGs was dependent on the functional status of the patient and increased as the patient’s condition worsened.

#### 3.4.1. Caregivers’ Health-Related Quality of Life

**Figure 3:**
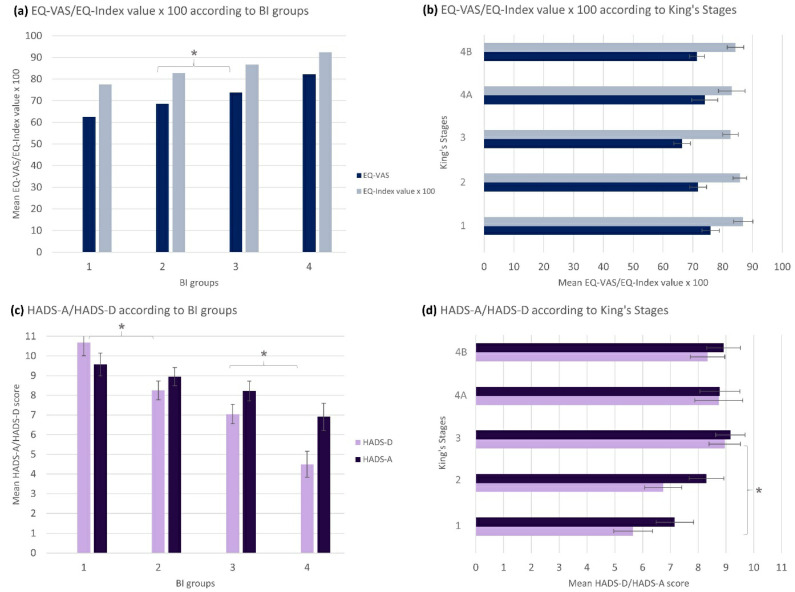
Caregivers’ health-related quality of life and anxiety/depression: Mean scores are presented with standard errors. Statistically significant findings: (**a**) EQ-VAS/EQ-5D-5L index value-BI groups (ANOVA: *p* < 0.001; KWT: *p* < 0.001; r_s_ = 0.317, *p* < 0.001, *n =* 243/ANOVA: *p* = 0.005; KWT: *p* = 0.001; r_s_ = 0.252, *p* < 0.001, *n =* 244); EQ-VAS/EQ-5D-5L index value-BI (r_p_ = 0.320, *p* < 0.001, *n =* 243/r_p_ = 0.258, *p* < 0.001, *n =* 244); (**b**) EQ-VAS/EQ-5D-5L index value-King’s Stages (no significant findings); EQ-VAS/EQ-5D-5L index value-ALSFRS-R (r_p_ = 0.278, *p* < 0.001, *n =* 238/r_p_ = 0.214, *p* = 0.001, *n =* 239); (**c**) HADS-D/HADS-A-BI groups (ANOVA: *p* < 0.001; KWT: *p* < 0.001; r_s_ = −0.376, *p* < 0.001, *n =* 240/ANOVA: *p* = 0.036; KWT: *p* = 0.03; r_s_ = −0.187, *p* = 0.004, *n =* 240); HADS-D/HADS-A-BI (r_p_ = −0.400, *p* < 0.001, *n =* 240/r_p_ = −0.200, *p* < 0.002, *n =* 240); (**d**) HADS-D/HADS-A-King’s Stages (ANOVA: *p* = 0.002; KWT: *p* = 0.001; r_s_ = 0.217, *p* = 0.001, *n =* 236/no significant findings); HADS-D/HADS-A-ALSFRS-R (r_p_ = −0.321, *p* < 0.001, *n =* 236/r_p_ = −0.193, *p* = 0.003, *n =* 236). * = post-hoc: *p* ≤ 0.05 between subgroups. Abbreviations: BI = Barthel Index; EQ-VAS = EuroQol visual analog scale; EQ-5D-5L index value x 100 = EuroQol Five Dimension Five Level Scale index value multiplied by 100; HADS-D = Hospital Anxiety and Depression Scale-depression subscale; HADS-A = Hospital Anxiety and Depression Scale-anxiety subscale.

The EQ-5D-5L measured the HRQoL. The EQ-5D-5L index value (mean = 0.845/1, SD = 0.196) was higher than the EQ-VAS (mean = 71.16/100, SD = 20.47). Both values were slightly lower than in the general German population (EQ-VAS mean = 79.45/100, SD = 17.05 [71], EQ-5D-5L index value mean = 0.88/1, SD = 0.18) [72]. We found statistically significant differences of the EQ-VAS/EQ-5D-5L index value between the different BI groups (Figure 3a). Additionally, there was a slight positive correlation between the HRQoL (EQ-VAS/EQ-5D-5L index value) and the ALSFRS-R (r_p_ = 0.278, *p* < 0.001/r_p_ = 0.214, *p* = 0.001) and the BI (r_p_ = 0.320, *p* < 0.001/r_p_ = 0.258, *p* < 0.001) as well as the derived BI groups. Nonetheless, no statistically significant correlation between the EQ-VAS nor the EQ-5D-5L index value and the King’s Stages was found (Figure 3b). Overall, this examination displayed that there was a significant influence of the patient’s functional status on the decrease in CG’s HRQoL that was depicted by proxy- and self-reported functional scales but not the King’s Stages.

#### 3.4.2. Caregivers’ Anxiety and Depression

With a median score of 8/21 in the HADS-D and 9/21 in the HADS-A, most CGs showed depression and anxiety. Again, we observed a correlation between the HADS-D/HADS-A scores and the different BI groups (Figure 3c). That implied that the scores increased towards more severe depression and anxiety with a lower BI group and thus more dependence of the patients. There was also a statistically significant difference of HADS-D, however not HADS-A, in dependence of the different King’s Stages (Figure 3d). Correspondingly, there was a statistically significant slight positive correlation between the HADS-D, however not the HADS-A, and the King’s Stages (r_s_ = 0.217, *p* = 0.001/r_s_ = 0.122, *p* = 0.061). In addition, negative correlations between depression and anxiety and the ALSFRS-R (r_p_ = −0.321, *p* < 0.001/r_p_ = −0.193, *p* = 0.003) as well as the BI (r_p_ = −0.400, *p* < 0.001/r_p_ = −0.200, *p* < 0.002) were observed. In summary, this analysis showed that loss of patients’ autonomy was a predictor of anxiety in CGs (median > 8), while depression was mainly influenced by the patients’ functional status and disease severity. 

#### 3.4.3. Caregivers’ Health Impairment

**Figure 4:**
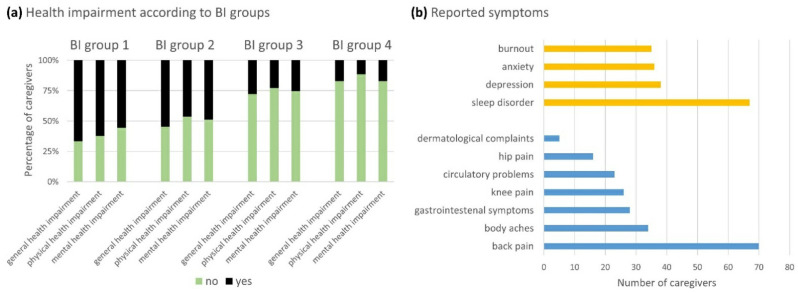
Caregivers’ health impairment: The caregivers were able to state if they felt health-impaired themselves due to caregiving (yes) or not (no) (**a**) and could differ between physical (blue) and mental (yellow) symptoms (**b**). Three adjacent columns in (**a**) are assigned to each BI group (groups 1–4). The extent of the colored sections represents the percentage of caregivers. We observed a statistically significant increase in the incidence of physical and mental health impairment according to the BI group (BI group 4 (=BI score 100 points = full independence) to BI group 1 (=BI score 0–20 points = full dependence)) and thus decreasing autonomy of the patients. Abbreviations: BI = Barthel Index.

Almost half (42.6%) of the CGs subjectively felt their own health impairment due to caregiving. While a smaller proportion of them reported only physical or mental health impairment (11.3% vs. 14.2%, respectively), 74.5% reported both (Table 2). We found statistically significant differences in the incidence of physical (χ^2^ = 32.67, *p* < 0.001; φ_c_ = 0.362, *p* < 0.001; *n =* 249) and mental health impairment (χ^2^ = 22.37, *p* < 0.001; φ_c_ = 0.300, *p* < 0.001; *n =* 249) between the BI groups (Figure 4a). By applying the same analyses to the King’s Stages, no statistically significant differences were found. While musculoskeletal system symptoms such as back pain, body aches, and knee and hip pain were the most frequently reported physical impairments, sleep disorder was the most frequent mental health complaint (Figure 4b).

#### 3.4.4. Caregivers’ Work Life Impairment

**Figure 5:**
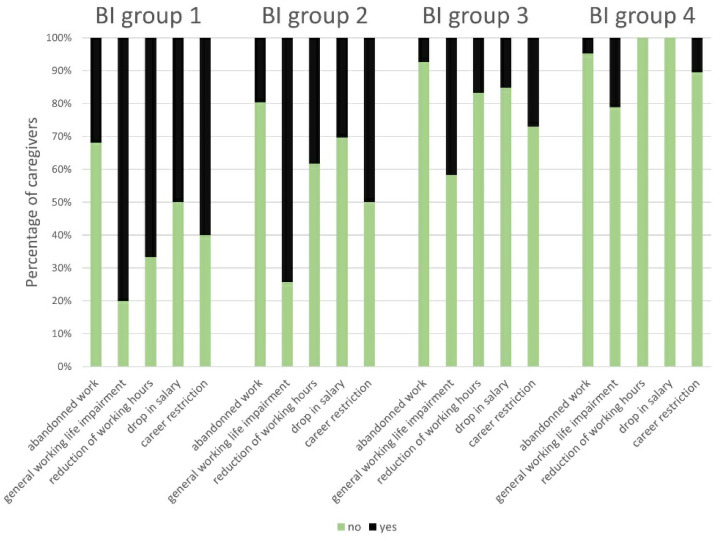
Impairment in caregivers’ work lives according to BI groups: The caregivers were able to state their work life impairment with “yes/no” in the following domains: abandoned work due to caregiving; general work life impairment; reduction of working hours; drop in salary; career restriction. Five adjacent columns are assigned to each BI group. One column corresponds to one domain. The extent of the colored sections represents the percentage of caregivers. There was a statistically significant correlation of the investigated domains to the BI groups. Abbreviations: BI = Barthel Index.

We excluded the CGs who were retired, unemployed or homemakers at the time of the survey from further analyses. The χ^2^ test showed statistically significant differences in the incidence of abandoning work between the several BI groups (χ^2^ = 9.05, *p* = 0.029; φ_c_ = 0.264, *p* = 0.029; *n =* 130) (Figure 5). Out of 110 working CGs, 59 (53.6%) stated to feel general impairment in their working lives and had to reduce their working time by 13.6 h/week on average, and 21 (19.1%) suffered from drop in their salary by 1209 € (Euro)/month gross on average. On closer examination, statistically significant differences between the BI groups were observed for the incidence of a general impairment in work life (χ^2^ = 20.40, *p* < 0.001; φ_c_ = 0.441, *p* < 0.001; *n =* 105); reduction of working hours (χ^2^ = 20.86, *p* < 0.001; φ_c_ = 0.454, *p* < 0.001; *n =* 101); drop in salary (χ^2^ = 12.84, *p* = 0.005; φ_c_ = 0.366, *p* < 0.001; *n =* 96) and career restriction (χ^2^ = 13.26, *p* = 0.004; φ_c_ = 0.355, *p* < 0.001; *n =* 105) (Figure 5). Therefore, CGs’ impairment in their work lives was associated with the patients’ functional status. No statistically significant results regarding the King’s Stages analyses were found.

### 3.5. Effect Comparison between Highly and Lowly Burdened Caregivers

In general, the total CG cohort showed a high burden with a median ZBI score of 26/88. By applying the cut-off at ≥ 24 points [59], we dichotomized the cohort into a lowly burdened (mean ZBI = 12.16/88, SD = 6.55, *n =* 107) and a highly burdened subgroup (mean ZBI = 37.47/88, SD = 9.16, *n =* 135). Using this cut-off of ≥ 24 points in the ZBI score, it was possible to compare the extent of the caregiving’s effects between the lowly and the highly burdened group. Here, we saw that a higher caregiver burden was associated with significantly worse HRQoL. In contrast, depression and anxiety were lower in the low burden group (Table 5). Moreover, the high burden group stated significantly more physical health and mental health impairment than the low burden group, confirming a worse health condition. According to the working abilities, the higher burdened CGs reported more cases of general work life impairment, a higher reduction of working hours, more frequent drops in salary, and career restrictions. In summary, the investigated impact parameters were associated with strain and thus a higher experienced burden worsened the CGs’ condition.

### 3.6. Gender-Specific Analysis

Gender-specific baseline characteristics of patients and their CGs are shown in Table 1 and Table 2. The female patients in our cohort had a stronger impaired functional status (mean BI = 50.97/100, SD = 31.26, *n =* 88) than the men (mean BI = 62.58/100, SD = 30.79, *n =* 157; U = 5415.00; Z = −2.81; *p* = 0.005; *n =* 245). When analyzing the female CG cohort’s care-related outcomes, it was noticeable, that caregiver burden, the DOC, HRQoL and depression were not significantly different to the men (Table 6). However, in contrast to the male cohort with a median HADS-A score of 7/21, female CGs with a median HADS-A score of 9/21 showed the presence of anxiety. Further, 16.6% of the healthily impaired female CGs specifically reported anxiety, compared to 9.3% of the healthily impaired male CGs. This finding was supported by a statistically significant correlation between the BI groups and the HADS-A in the female CGs (r_s_ = −0.280, *p* < 0.001, *n =* 169), and the simultaneously absent correlation in the male CG cohort (r_s_ = −0.007, *p* = 0.957, *n =* 71). In addition, further analyses regarding health and working life impairment did not show any significant results (Table 6). Figure 2b compares the self-rated general impairment in daily routine between male and female CGs. No significant differences in any domain were observed, except a statistical tendency (χ2 = 7.66; φ_c_ = 0.178, *p* = 0.054; *n =* 249) in the domain “time”, indicating that both male and female CGs experienced their daily routine impairments in a similar way.

### 3.7. Correlation of Parameters

**Figure 6:**
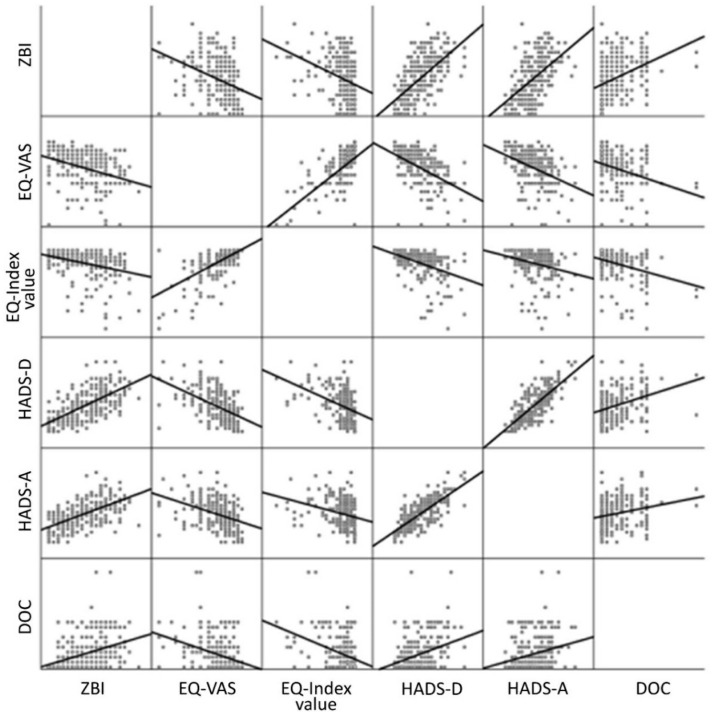
Correlation of parameters: Statistically significant correlations: ZBI-DOC (r_p_ = 0.379, *p* < 0.001, *n =* 242); ZBI-HADS-D (r_p_ = 0.627, *p* < 0.001, *n =* 234); ZBI-HADS-A (r_p_ = 0.550, *p* < 0.001, *n =* 234); ZBI-EQ-VAS/EQ-5D-5L index value (r_p_ = −0.362, *p* < 0.001, *n =* 237/r_p_ = −0.315, *p* < 0.001, *n =* 238); HADS-D-HADS-A (r_p_ = 0.745, *p* < 0.001, *n =* 240), HADS-D-DOC (r_p_ = 0.332, *p* < 0.001, *n =* 240); HADS-D-EQ-VAS/EQ−5D-5L index value (r_p_ = −0.488, *p* < 0.001, *n =* 238/r_p_ = −0.399, *p* < 0.001, *n =* 238); HADS-A-DOC (r_p_ = 0.230, *p* < 0.001, *n =* 240), HADS-A-EQ-VAS/EQ-5D-5L index value (r_p_= −0.386, *p* < 0.001, *n =* 238/r_p_ = −0.272, *p* < 0.001, *n =* 238); DOC-EQ-VAS/EQ-5D-5L index value (r_p_= −0.371, *p* < 0.001, *n =* 243/r_p_ = −0.375, *p* < 0.001, *n =* 244). Abbreviations: ZBI = Zarit Burden Interview; EQ-VAS = EuroQol visual analog scale; EQ-5D-5L index value = EuroQol Five Dimension Five Level Scale index value; HADS-D = Hospital Anxiety and Depression Scale-depression subscale; HADS-A = Hospital Anxiety and Depression Scale-anxiety subscale; DOC = duration of caregiving.

Figure 6 shows a correlation matrix of burden (ZBI score), depression (HADS-D score), anxiety (HADS-A score), HRQoL (EQ-VAS/EQ-5D-5L index value) and DOC. Several correlations between the parameters were observed. The statistically significant positive correlations between the ZBI and the HADS-D scores, as well as the HADS-A score and a negative correlation between the ZBI score and the EQ-VAS/EQ-5D-5L index value, confirmed the findings in the effect comparison between the highly and the lowly burdened CGs (see Section 3.5). Additionally, a statistically significant positive correlation was found between the HADS-D and the HADS-A scores, besides a negative correlation of both to the EQ-VAS/EQ-5D-5L index value, which indicated that depression, anxiety and lower HRQoL were interrelated. Nevertheless, this result has to be interpreted with caution as the overlap between the EQ-5D-5L domain anxiety/depression and the HADS might favor this effect. Furthermore, there was a statistically significant positive correlation between the DOC and the ZBI score, as well as to the HADS-D and HADS-A scores, compared to a negative correlation to the EQ-VAS/EQ-5D-5L index value. That indicated that an increased time spent with the patient went along with a higher caregiver burden, incidence of depression and anxiety and a lower HRQoL. Overall, due to existing correlations between all parameters, the caregiving strain appeared to result from complex interactions between different factors. 

### 3.8. Caregivers’ Wishes and Treatment Satisfaction

At the end of the study questionnaire, CGs were given the opportunity to express their wishes and needs in a text field by using their own words. For qualitative analysis, the answers were categorized according to the topics they most frequently addressed: the need for psychological support for caregivers (31%), the bureaucracy of the health insurance as well as its hurdles in the supply, e.g., with medical devices, was a major obstacle (22%). Additionally, CGs caring for their partners wished for a joint rehabilitation with the patient (17%). Furthermore, financial support (9%) and a greater relief by care services were requested (15%). Some CGs felt insecure in their nursing and requested classes or teaching courses to improve their caregiving skills (6%). 

The satisfaction level of the CGs regarding their own (medical) care among the investigated population was modest (arithmetic mean = 2.18, *n =* 243, on a Likert scale 1–4: 1 = ”very satisfied” (20.2%, *n =* 49); 2 = ”satisfied” (48.6%, *n =* 118); 3=”not satisfied” (27.6%, *n =* 67); 4=”not satisfied at all”(3.7%, *n =* 9)). 

## 4. Discussion

This study is the first to specifically investigate the general burden on people caring for patients with ALS in Germany. For assessment of the informal caregivers’ burden, the ZBI score, the DOC and several self-reporting tools were used that reflect personal demands, mental and physical health status, and quality of life. In particular, these measures were analyzed with regard to the functional status of those ALS patients the CGs were caring for. 

Overall, CGs of ALS-affected persons experienced a high burden, more than half of them (55.8%) even belonged to a highly burdened CG group per definition (ZBI score ≥ 24; mean 37.5/88). However, the lowly burdened CGs should not be understood as not affected, but as potentially receptive for increasing burden in future with decreasing patient’s autonomy. The main study results showed an increasing burden (ZBI score) and DOC with patients’ worsening functional status and disease severity. Patients’ use of a wheelchair and need for the permanent (24 h) supervision were the main determinants of caregiver burden and DOC. While mental health impairment of the CG affected burden, physical health impairment affected the DOC. The incidence of general impairment in everyday life, anxiety, depression, health impairment and work life impairment increased while the CGs’ HRQoL decreased with decreasing patient autonomy and were associated with higher burden. 

With a mean ZBI of 26.28/88 (mean ALSFRS-R = 31.39/48, *n =* 242), our cohort of CGs was severely burdened as a result of caring for ALS patients, which is in line with previous results presented by Burke et al. (ZBI 26.74/88, mean ALSFRS-R = 32.94/48, *n =* 85) [27], Galvin et al. (ZBI 27.1/88, *n* = 81) [26], or longitudinal approaches using machine learning as used by Antoniadi et al. (ZBI 26.9/88, mean ALSFRS-R = 32.8/48, *n =* 90) [19]. Caregiver burden appears to be similar throughout these studies due to the comparable functional status of the study populations and is only marginally influenced by the slightly different data collection methods (questionnaires and interviews). Therefore, our results confirm the impact of the patient’s functional status on her/his CG’s burden. Compared to other neuromuscular disorders (NMD), we estimated a caregiver burden comparable to Duchenne muscular dystrophy in a study cohort from Germany, UK, Italy and the US (mean ZBI score = 29/88, *n =* 770) [73] and a lower burden compared to spinal muscular atrophy (mean = 31.9/88, *n =* 68) [74].

Previous studies do not agree whether patients’ disease progression has an impact on caregiver’s burden [13,32,75] or not [27,31,37]. In the present study, patients’ functional status (BI, ALSFRS-R, BI group) and their disease severity (King’s Stages) were major influencing factors on caregiver’s burden and the DOC, since a lower functional status and thus a higher disease severity demanded more extensive care. In addition, it became apparent that wheelchair dependency, the need for ventilation support [6,28] and the need for permanent supervision had a large impact on the general burden and the DOC, as these were indicators for a less independent patient. Previous studies additionally identified neurobehavioral ALS symptoms [76], patient’s behavioral changes [31], patient’s apathy [35,77], disinhibition [35] and executive dysfunction [35] to be predictors for higher caregiver burden, while, spirituality and existential wellbeing [78], social support [75], as well as mindfulness [30] were described as protective factors. The latter was also been shown to be inversely associated with caregiver burden in e.g., Parkinson’s disease [79]. In our study, the distinction between mental and physical health allowed us to identify an increase of DOC by CGs’ physical but not mental health impairment. This circumstance may be seen as a vicious cycle, as extensive caregiving leads to physical health impairments [80], which worsened the CG’s nursing abilities and thus further increased the DOC. In contrast, our study showed that mental health impairment, but not physical health impairment, had an impact on the perceived burden of care. These observations distinguish between two distinct types of strain: somatic complaints due to physical stress and mentally experienced burden. 

It is noticeable that nearly half of the CGs (42.6%) reported their own health impairment due to caregiving. Less than 20% of these CGs reported either mental or physical impairments, as most of them suffered from both. Furthermore, the incidence of CGs’ health impairment was related to the patients’ functional status. Due to the physical exertion during nursing, musculoskeletal symptoms were most commonly reported. Similar to CGs of Parkinson’s disease patients, sleep disorders and depression were also frequently indicated. Herein, correlations between CGs’ quality of sleep and patients’ quality of sleep, depression, and quality of life were the explanation for CG’s sleep disorder. [81].

One of the negative consequences of caregiving was the CGs’ decreased HRQoL. CGs’ self-reported current health state as reflected by the EQ-VAS was lower than the calculated EQ-5D-5L index value (mean 71.16/100 vs. 0.845/1.0). However, this circumstance is not extraordinary since the EQ-VAS is influenced by many other factors like age, education, ethnicity, smoking and perceived control [82]. It was not surprising that CGs showed a lower HRQoL than the general German population (79.45/100, [71]. Compared to previous reports, the mean EQ-VAS in our ALS cohort was slightly higher than reported by Sandstedt et al. (66/100, *n* = 49) [34], however, this cohort’s disease severity was not described in the paper. The mean EQ-5D-5L index value of CGs in Duchenne muscular dystrophy was reported in a similar range (mean EQ-5D-5L index value = 0.81) [73] to ALS. In our study, we were not able to show that the CGs’ HRQoL decreased with the patients’ disease severity (as defined by King’s Stages), which is comparable to previous findings [15,34], although we found the patients’ functional status to have an influence on the CGs’ HRQoL (BI and BI group). Additionally, similar to previous research [33], the experienced burden also had an impact on HRQoL. Further, interrelations to other parameters such as anxiety and depression were found, as suspected before [16,17]. In contrast to the CGs, previous research showed the absence of loss in quality of life with a decline in physical health in patients [83].

Our CG cohort showed lower anxiety in relation to the disease severity of patients (mean HADS-A = 8.55/21; mean ALSFRS-R = 31.39/48, *n =* 240), than previous study results from Burke et al., (mean HADS-A = 9.42/21; mean ALSFRS-R = 32.94; *n =* 85) [27] and Galvin et al. (mean HADS-A = 9.6/21; *n =* 81) [26]. In contrast, our cohort appeared to be more depressed (mean HADS-D = 7.80/21; mean ALSFRS-R = 31.39/48, *n =* 240) in comparison to Burke et al. (mean HADS-D = 5.73/21; mean ALSFRS-R = 32.94; *n =* 85) [27] and Galvin et al., (mean HADS-D = 5.9/21; *n =* 81) [26]. Compared to analyses in Parkinson’s disease, our cohort revealed higher levels of anxiety and depression (mean HADS-A = 7.4/21; mean HADS-D = 4.3/21; *n =* 175) [56]. A possible explanation may be the differences in disease progression and treatment possibilities between ALS and Parkinson’s disease [84]. Unlike previous research [37,85], we observed that the level of depression and anxiety was related to the patient’s functional status; however, only depression significantly increased with disease severity. Similar to previous findings [27,85], correlations between anxiety, depression and caregiver burden were also verified. 

In our gender-specific analysis, we investigated possible differences in burden and impairments between caregiving women and men. Unlike in previous research by Tramonti et al. (mean Caregiver Burden Inventory 31.25 in men vs. 38.64 in women; *n =* 89) [36] we cannot confirm a difference in burden perception between the two genders (mean ZBI of 27.60/88 in men vs. 25.68/88 in women; *n =* 242). However, female CGs showed a presence of anxiety in contrast to men (mean HADS-A of 7.69/21 in men vs. 8.91/21 in women; *n =* 240). Besides this finding, no significant differences were observed in any other gender analysis, especially in the previously suspected higher prevalence of depression in female CGs [37].

Our analysis of CGs’ professional life situations implied that increasing demand for care may lead to the abandonment of employment since this was seen less frequently in higher (=better) BI scores. General work life impairment was also related to patients’ functional status. Unfortunately, it seemed to be a downward spiral, in which CGs were forced to reduce working time to assure patients’ care, causing a drop in salary and career restrictions. Moreover, additional disease costs emerge, caused by loss of productivity and additional need for treatment of the affected CG [7]. Therefore, it is important to gather data about work life impairment in caregiver burden analyses. 

As other studies have attempted, we examined the previously suspected interrelation between various negative effects [16] and investigated correlations between caregiver burden, DOC, anxiety, depression and HRQoL. Our correlation matrix proved that anxiety, depression, caregiver burden, HRQoL and DOC are interrelated, meaning that these effects rarely appear individually. Thus, it is not easy to form a causal chain of these parameters as it was tried in studies before, e.g., defining depression or anxiety as a predictor of burden [27]. Indeed, it further has to be discussed, if an existing depression may amplify the experienced burden, but also if the caregiving burden may have led to depression and lowered HRQoL. More precisely, the caregiving’s aftermath has to be viewed as a multifactorial construct of effects, built by the constant confrontation with the inevitable exacerbation of a close relative’s condition. 

The poor care applied to the informal caregivers could explain the modest satisfaction level (arithmetic mean = 2.18, *n =* 243, on a 1–4 Likert scale), which was reflected in their wishes and needs. Especially the lack of psychological support could be responsible for the present caregiver burden. Moreover, the reduction of the bureaucracy of health insurance as well as its hurdles in the supply, e.g., with medical devices, was a major wish of CGs. The description of CGs’ wishes was another aim of this study. Altogether, this shows that the situation of CGs needs more attention with, apart from their own medical and psychological care, more support in care and patient management issues.

Our study’s strength is the multi-center and nation-wide study cohort, which, to our knowledge, to date is the largest reported ALS patient and CG cohort in Germany and one of the greatest internationally. Furthermore, our participants were nearly evenly split across German states corresponding to the general population’s distribution. In addition, the variety of applied tools provided us with a deeper insight into the topic of caregiver burden in ALS. Although the assessment of the patient’s functional status with the BI and the ALSFRS-R seems to be redundant, we felt it necessary to report the CG’s (proxy-reported) and the patient’s self-reported perspectives. Herein, we observed a higher correlation of the BI to the general burden and all consequent outcomes, which may favor the CG’s perspective and the assessment of especially nursing-related impairments in daily routine. Since the presence of fronto-temporal dementia was not assessed, our study is not able to identify this condition as a potential amplifier of burden. Our questionnaire was rather detailed with more than 100 questions for the patient, and more than 50 for the CG, which may result in selection bias towards more motivated attendees, though the cohort was very well distributed regarding the BI groups. The main predictor in most of our investigations was the patients’ functional status (BI), which also provided more unequivocal analyses than the disease severity (King’s Stages). A possible explanation could be seen in the reporting methods: ALSFRS-R was patient-reported, as was consequently the derived King’s Stages, while the BI was proxy-reported by the CG. There may be a discrepancy between the patient’s subjective evaluation and the CG’s proxy-estimation of the situation, which can lead to different assessments of the actual condition, causing a potential report bias. This phenomenon was already observed in Parkinson’s disease, as CGs tended to assess higher severity than patients [86]. Also, in ALS, it is known, that caregivers tend to overestimate patients’ distress and underestimate their quality of life [87,88]. Longitudinal studies, which do allow depicting the full picture of the patients’ disease progression and caregiver burden over a course of time, may help to further address this issue. Moreover, machine-learning approaches for a possible early diagnosis [19] combined with EMA (ecological momentary assessments) could provide a method of intervention [89].

To summarize, our study not only confirmed but also highlighted previous findings in this field in more detail. Firstly, caregiver burden in ALS is a serious issue, which rises with the progressive loss of the patient’s autonomy. Further, the strain of caregiving is accompanied by health impairments and results in lower HRQoL on the CGs’ side. CGs’ work life impairment has to be considered as a potential socioeconomic burden. Furthermore, clinicians should be aware of possible depression as well as anxiety (the latter with occurrence mainly in female CGs), and try to prevent and intervene early. Regular medical investigations of CGs’ health states seem to be mandatory early in the disease course in order to be able to provide the necessary support and therapies timely. This is additionally emphasized by the suspicion that a high caregiver burden also may result in poorer patient care. Therefore, this responsibility should not be left on the CGs’ alone, but societal health strategies and universal standards of care should be implemented, as already done in other diseases [90,91]. Moreover, health authorities and health insurance companies have to facilitate application processes and provide quick and straightforward help. Finally yet importantly, the caregiving’s aftermath has to be seen as a multifactorial construct of interrelated effects which define caregiving’s hardship in ALS.

## 5. Conclusions

The care of patients with ALS causes a high individual burden on the lives of their relatives, respectively CGs. Additionally, they face significant impairment of their own mental and physical health and severe restrictions regarding work life, income, and socioeconomic status. Aside from new disease-modifying therapeutic options, better concepts, as requested by the CGs themselves, as well as standards of care should be established that include the CGs to improve their situation on the one hand and patient care (indirectly) on the other hand. Clinicians therefore should elucidate early, include the CGs in the treatment from the very beginning and health authorities have to abolish existing obstacles and implement standardized support programs.

## Figures and Tables

**Table 1:** Patients’ characterization. This table shows the patients’ characteristics, their functional status (BI, ALSFRS-R, BI groups) and their disease stages (King’s Stages). Due to rounding, the percentage might not add up to 100 exactly. * = Since some data (e.g., the sex of four patients) was unknown, not all numbers add up in total and result in different *n*. Abbreviations: *n =* number; IQR = interquartile range; SD = standard deviation; ALS = Amyotrophic Lateral Sclerosis; PEG = percutaneous endoscopic gastrostomy; BI = Barthel Index; ALSFRS-R = Revised Amyotrophic Lateral Sclerosis Functional Rating Scale. Median and IQR are presented in bold.

Patients’ Characterization (*n* = All, *n =* Men, *n =* Women); Score’s Range	All Absolute Number, Percent or Median (IQR)/Mean (SD)	Men Absolute Number, Percent or Median (IQR)/Mean (SD)	Women Absolute Number, Percent or Median (IQR)/Mean (SD)
Number *	249, 100	157, 64.1	88, 35.9
Age, years (*n* = 245, *n =* 157, *n =* 88)	**65 (16)**/64.19 (11.67)	**64 (16)**/63.59 (12.25)	**65.5 (15.25)**/65.27 (10.53)
Marital status (*n* = 243, *n =* 156, *n =* 87)			
• Single	9, 3.7	5, 3.2	4, 4.6
• Living in a partnership	15, 6.2	9, 5.8	6, 6.9
• Married	202, 83.1	139, 89.1	63, 72.4
• Divorced	11, 4.5	3, 1.9	8, 9.2
• Widowed	6, 2.5	0, 0.0	6, 6.9
Housing situation (*n* = 233, *n =* 151, *n =* 82)			
• With the family/partner	212, 91.0	142, 94.0	70, 85.4
• Alone	16, 6.9	5, 3.3	11, 13.4
• Assisted living	2, 0.9	2, 1.3	0, 0.0
• Nursing home	3, 1.3	2, 1.3	1, 1.2
Employment (*n* = 222, *n =* 143, *n =* 79)			
• Working	31, 14.0	34, 23.8	26, 32.9
• Employment no longer possible due to ALS	60, 27.0	25, 17.5	6, 7.6
• Retired due to age, unemployed, homemaker	131, 59.0	84, 58.7	47, 59.5
Use of wheelchair (*n* = 249, *n =* 157, *n =* 88)	120, 48.2	69, 43.9	49, 55.7
Use of home ventilator support (*n* = 249, *n =* 157, *n =* 88)	53, 21.3	37, 23.6	16, 18.2
Use of PEG (*n* = 249, *n =* 157, *n =* 88)	38, 15.3	19, 12.1	18, 20.5
Permanent supervision necessary (*n* = 221, *n =* 143, *n =* 78)	92, 41.6	46, 32.2	46, 59.0
BI score (*n* = 249, *n =* 157, *n =* 88); 0–100	**60 (60)**/58.63 (31.23)	**65 (60)**/62.58 (30.79)	**45 (58.75)**/50.97 (31.26)
BI groups (*n* = 249, *n =* 157, *n =* 88)			
• 1 (0–20)	45, 18.1	23, 14.6	22, 25.0
• 2 (21–60)	86, 34.5	51, 32.5	34, 38.6
• 3 (61–99)	83, 33.3	55, 35.0	25, 28.4
• 4 (100)	35, 14.1	28, 17.8	7, 8.0
ALSFRS-R score (*n* = 244, *n =* 157, *n =* 87); 0–48	**32.5 (13.75)**/31.39 (9.52)	**34 (16)**/32.38 (9.50)	**30 (13)**/29.61 (9.33)
King’s Stages (*n* = 244, *n =* 157, *n =* 87)			
• 1	39, 16.0	31, 19.7	8, 9.2
• 2	53, 21.7	35, 22.3	18, 20.7
• 3	66, 27.0	36, 22.9	30, 34.5
• 4A	29, 11.9	15, 9.6	14, 16.1
• 4B	57, 23.4	40, 25.5	17, 19.5

**Table 2:** Caregivers’ characterization. This table shows the caregivers’ characteristics, their care-related burden (ZBI score), their HRQoL (EQ-VAS score/EQ-5D-5L index value), and their depression and anxiety (HADS-D/HADS-A scores). Due to rounding, the percentage might not add up to 100 exactly. * = Since some data (e.g., the ZBI of seven CGs) was unknown, not all numbers add up in total and result in different *n*. Abbreviations: *n =* number; CGs = caregivers; IQR = interquartile range; SD = standard deviation; ALS = Amyotrophic Lateral Sclerosis; DOC = duration of caregiving; ZBI = Zarit Burden Interview; EQ-VAS = EuroQol visual analog scale; EQ-5D-5L index value = EuroQol Five Dimension Five Level Scale index value; HADS-D = Hospital Anxiety and Depression Scale-depression subscale; HADS-A = Hospital Anxiety and Depression Scale-anxiety subscale. Median and IQR are presented in bold.

Caregivers’ Characterization (*n* = All, *n =* Men/Male CGs, *n =* Women/Female CGs); Score’s Range	All Absolute Number, Percent or Median (IQR)/Mean (SD)	Male CGs Absolute Number, Percent or Median (IQR)/Mean (SD)	Female CGs Absolute Number, Percent or Median (IQR)/Mean (SD)
Number *	249, 100.0	75, 30.1	174, 69.9
Age, years (*n* = 248, *n =* 75, *n =* 173)	**61(16.75)**/59.84 (13.77)	**65 (16)**/64.23 (13.47)	**60 (16)**/57.94 (13.52)
Marital status (*n* = 249, *n =* 75, *n =* 174)			
• Single	9, 3.6	5, 6.7	4, 2.3
• Living in a partnership	23, 9.2	10, 13.3	13, 7.5
• Married	214, 86.0	60, 80.0	154, 88.5
• Divorced	2, 0.8	0, 0.0	2, 1.1
• Widowed	1, 0.4	0, 0.0	1, 0.6
Relation to the patient (*n* = 249, *n =* 75, *n =* 174)			
• Spouse	207, 83.1	67, 89.3	140, 80.5
• Parent	17, 6.8	5, 6.7	12, 6.9
• Sibling	8, 3.2	0, 0.0	8, 4.6
• Child	15, 6.0	3, 4.0	12, 6.9
• Friend	2, 0.8	0, 0.0	2, 1.1
Employment (*n* = 249, *n =* 75, *n =* 174)			
• Working	110, 44.2	25, 33.3	85, 48.9
• Employment no longer possible due to patient’s ALS	20, 8.0	7, 9.3	13, 7.5
• Retired, unemployed, homemaker	119, 47.8	43, 57.3	76, 43.7
Health impairment due to caregiving (*n* = 249, *n =* 75, *n =* 174)	106, 42.6	32, 42.7	74, 42.5
• Only physical impairment	12, 11.3	4, 12.5	8, 10.8
• Only mental impairment	15, 14.2	5, 15.6	10, 13.5
• Physical and mental impairment	79, 74.5	23, 71.9	56, 75.7
DOC, hours per day (*n* = 249, *n =* 75, *n =* 174)	**3 (6)**/3.85 (4.25)	**3 (6)**/4.31 (4.03)	**2.5 (5)**/3.65 (4.34)
ZBI score (*n* = 242, *n =* 74, *n =* 168); 0–88	**26 (25)**/26.28 (14.97)	**25 (26.25)**/27.60 (15.25)	**26 (25)**/25.68 (14.86)
EQ-VAS score (*n* = 243, *n =* 73, *n =* 170); 0–100	**75 (28)**/71.16 (20.47)	**75 (20)**/69.27 (19.29)	**75 (30)**/71.97 (20.96)
EQ-5D-5L index value (*n* = 244, *n =* 73, *n =* 171); −0.205–1.0	**0.909 (0.193)**/0.845 (0.196)	**0.909 (0.102)**/0.849 (0.164)	**0.909 (0.193)**/0.844 (0.209)
HADS-D score (*n* = 240, *n =* 71, *n =* 169); 0–21	**8 (7)**/7.80 (4.68)	**8 (7)**/7.93 (4.47)	**7 (7)**/7.75 (4.77)
HADS-A score (*n* = 240, *n =* 71, *n =* 169); 0–21	**9 (6)**/8.55 (4.28)	**7 (6)**/7.69 (3.92)	**9 (6.5)**/8.91 (4.38)

**Table 3:** Influencing factors on caregiver burden (ZBI score): The ZBI score constituted the dependent variable and patient-related as well as caregiver-related parameters were analyzed as possibly influencing independent variables. This multiple linear regression analysis showed a statistically significant influence of the CG’s mental health impairment, patient’s wheelchair use, patient’s age, and the necessity of permanent attendance of a helper/supervisor on the ZBI score. The model was adjusted for statistical outliers. The results are arranged by *p*-values. Abbreviations: ZBI = Zarit Burden Interview; CI = confidence interval; PEG = percutaneous endoscopic gastrostomy.

Variable	Change in ZBI	95% CI		*p*-Value
Mental health impairment of the caregiver	11.36	6.84	15.87	<0.001
Use of wheelchair	9.30	5.94	12.66	<0.001
Patient’s age	−0.24	−0.37	−0.11	<0.001
Permanent supervision necessary	5.01	1.63	8.38	0.004
Use of PEG	3.80	−0.25	7.86	0.066
Use of home ventilator support	2.77	−0.97	6.51	0.146
Physical health impairment of caregiver	1.96	−2.64	6.55	0.402

**Table 4:** Influencing factors on DOC: The DOC score (in hours per day) constituted the dependent variable and patient-related as well as caregiver-related parameters were analyzed as possibly influencing independent variables. This multiple linear regression analysis showed a statistically significant influence of wheelchair use, the necessity of permanent supervision, physical health impairment of the caregiver himself, and the use of ventilation support on the DOC. The model was adjusted for statistical outliers. The results are arranged by *p*-values. Abbreviations: DOC = duration of caregiving; CI = confidence interval.

Variable	Change in DOC	95% CI		*p*-Value
Use of wheelchair	2.29	1.22	3.36	<0.001
Permanent supervision necessary	1.86	0.78	2.93	0.001
Physical health impairment of the caregiver	1.62	0.15	3.09	0.031
Use of home ventilator support	1.32	0.12	2.52	0.031
Patient’s age	0.03	−0.02	0.07	0.214
Mental health impairment of the caregiver	0.85	−0.59	2.30	0.246

**Table 5:** Effect comparison between the high and the low burden group: The caregiver burden had effects on the CG’s HRQoL, depression and anxiety, their physical and mental health impairment, general work life impairment, career restrictions, reduction of working hours, and drop in salary. Abbreviations: ZBI = Zarit Burden Interview; *n =* number; EQ-VAS = EuroQol visual analog scale; SD = standard deviation; HADS-D = Hospital Anxiety and Depression Scale-depression subscale; HADS-A = Hospital Anxiety and Depression Scale-anxiety subscale.

		Low Burden Group (ZBI Score < 24), *n =* 107	High Burden Group (ZBI Score ≥ 24), *n =* 135	Low vs. High Burden Group
**EQ-VAS**, range 0–100	Mean (SD) Mann–Whitney test	78.85 (18.25), *n =* 103	64.93 (20.45), *n =* 134	U = 3750.50; Z = −6.05; *p* < 0.001; *n =* 237
**EQ-5D-5L index value**, range 0–1	Mean (SD) Mann–Whitney test	0.905 (0.128), *n =* 104	0.796 (0.230), *n =* 134	U = 4306.50; Z = −5.01; *p* < 0.001; *n =* 238
**HADS-D score**, range 0–21	Mean (SD) Mann–Whitney test	4.78 (3.47), *n =* 101	10.05 (4.24), *n =* 133	U = 2285.00; Z = −8.66; *p* < 0.001; *n =* 234
**HADS-A score**, range 0–21	Mean (SD) Mann–Whitney test	6.02 (3.42), *n =* 101	10.52 (3.77), *n =* 133	U = 2580.00; Z = −8.09; *p* < 0.001; *n =* 234
**Physical health impairment**	Relative frequency χ2-test; Cramér’s V	11.2%, *n =* 107	57.8%, *n =* 135	χ2 = 55.40; φ_c_ = 0.478, *p* < 0.001; *n =* 242
**Mental health impairment**	Relative frequency χ2-test; Cramér’s V	11.2%, *n =* 107	60.0%, *n =* 135	χ2 = 60.04; φ_c_ = 0.498, *p* < 0.001; *n =* 242
**General work life impairment**	Relative frequency χ2-test; Cramér’s V	25.0%, *n =* 44	78.0%, *n =* 59	χ2 = 28.61; φ_c_ = 0.527, *p* < 0.001; *n =* 103
**Career restrictions**	Relative frequency χ2-test; Cramér’s V	13.6%, *n =* 44	51.7%, *n =* 58	χ2 = 15.89; φ_c_ = 0.395, *p* < 0.001; *n =* 102
**Reduction of working hours**	Relative frequency χ2-test; Cramér’s V	11.6%, *n =* 43	60.0%, *n =* 55	χ2 = 9.73; φ_c_ = 0.315, *p* = 0.001; *n =* 98
**Drop in salary**	Relative frequency χ2-test; Cramér’s V	12.5%, *n =* 40	30.2%, *n =* 53	χ2 = 4.01; φ_c_ = 0.209, *p* = 0.043; *n =* 93

**Table 6:** Effect comparison between men and women: Besides the difference in anxiety, no statistical differences were found. Abbreviations: CGs = caregivers; *n =* number; ZBI = Zarit Burden Interview; SD = standard deviation; DOC = duration of caregiving; EQ-VAS = EuroQol visual analog scale; HADS-D = Hospital Anxiety and Depression Scale-depression subscale; HADS-A = Hospital Anxiety and Depression Scale-anxiety subscale.

Effect Comparison between the Female and Male CG Cohort				
		Male CGs, *n =* 75	Female CGs, *n =* 174	Male vs. female CGs
**ZBI**, range 0–88	Mean (SD) Mann–Whitney test	27.60 (15.25), *n =* 74	25.68 (14.86), *n =* 168	U = 5825.50; Z = −0.78; *p* = 0.436; *n =* 242
**DOC**	Mean (SD) Mann–Whitney test	4.31 (4.03), *n =* 75	3.65 (4.34), *n =* 174	U = 5692.00; Z = −1.62; *p* = 0.105; *n =* 249
**EQ-VAS**, range 0–100	Mean (SD) Mann–Whitney test	69.27 (19.29), *n =* 73	71.97 (20.96), *n =* 170	U = 5463.50; Z = −1.48; *p* = 0.138; *n =* 243
**EQ-5D-5L index value**, range 0–1	Mean (SD) Mann–Whitney test	0.849 (0.164), *n =* 73	0.844 (0.209), *n =* 171	U = 5686.50; Z = −0.75; *p* = 0.457; *n =* 244
**HADS-D score**, range 0–21	Mean (SD) Mann–Whitney test	7.93 (4.47), *n =* 71	7.75 (4.77), *n =* 169	U = 5744,50; Z = −0.52; *p* = 0.603; *n =* 240
**HADS-A score**, range 0–21	Mean (SD) Mann–Whitney test	7.69 (3.92), *n =* 71	8.91 (4.38), *n =* 169	U = 5059.00; Z = −1.92; *p* = 0.055; *n =* 240
**Physical health impairment**	Relative frequency χ2-test; Cramér’s V	34.7%, *n =* 75	37.4%, *n =* 174	χ2 = 0.16; φ_c_ = 0.026, *p* = 0.686; *n =* 249
**Mental health impairment**	Relative frequency χ2-test; Cramér’s V	36.0%, *n =* 75	38.5%, *n =* 174	χ2 = 0.14; φ_c_ = 0.024, *p* = 0.708; *n =* 249
**General work life impairment**	Relative frequency χ2-test; Cramér’s V	45.8%, *n =* 24	56.8%, *n =* 81	χ2 = 0.90; φ_c_ = 0.092, *p* = 0.344; *n =* 105
**Career restrictions**	Relative frequency χ2-test; Cramér’s V	24.0%, *n =* 25	40.0%, *n =* 80	χ2 = 2.11; φ_c_ = 0.142, *p* = 0.146; *n =* 105
**Reduction of working hours**	Relative frequency χ2-test; Cramér’s V	33.3%, *n =* 24	24.7%, *n =* 77	χ2 = 0.70; φ_c_ = 0.083, *p* = 0.403; *n =* 101
**Drop in salary**	Relative frequency χ2-test; Cramér’s V	19.0%, *n =* 21	22.7%, *n =* 75	χ2 = 0.126; φ_c_ = 0.036, *p* = 0.723; *n =* 96

## Data Availability

The data presented in this study are available on request from the corresponding author. The data are not publicly available due to privacy and ethical reasons.

## Appendix A

**Table A1:** Regional distribution of ALS patients included in this study compared with the regional distribution by state of the general population in Germany. The distribution of our patients roughly matches the regional population distribution by state in Germany. Due to rounding, percentages do not add up to exactly 100. Abbreviations: ALS, amyotrophic lateral sclerosis; *n*, number.

State	*n* Included in Study (Percentage)	Total Population of the State, 2019 [in Thousands] (Percentage) [67]	Study Population’s Difference (Percentage Points)
Lower Saxony	92 (36.9%)	7994 (9.7%)	+27.2
North Rhine-Westphalia	62 (24.9%)	17,947 (21.6%)	+3.3
Bavaria	46 (18.5%)	13,125 (15.8%)	+2.7
Saxony	15 (6.0%)	4072 (4.9%)	+1.1
Baden-Wuerttemberg	8 (3.2%)	11,100 (13.3%)	−10.1
Hesse	6 (2.4%)	6288 (7.6%)	−5.2
Schleswig-Holstein	6 (2.4%)	2904 (3.5%)	−1.1
Saxony-Anhalt	5 (2.0%)	2195 (2.6%)	−0.6
Rhineland-Palatinate	4 (1.6%)	4094 (4.9%)	−3.3
Bremen	2 (0.8%)	681 (0.8%)	0
Mecklenburg-Western Pomerania	1 (0.4%)	1608 (1.9%)	−1.5
Brandenburg	1 (0.4%)	2522 (3.0%)	−2.6
Thuringia	1 (0.4%)	2133 (2.6%)	−2.2
Berlin	0 (0.0%)	3669 (4.4%)	−4.4
Hamburg	0 (0.0%)	1847 (2.2%)	−2.2
Saarland	0 (0.0%)	987 (1.2%)	−1.2
